# Perioperative active warming strategies in children: a protocol for a multicentre, prospective, randomized controlled trial

**DOI:** 10.3389/fped.2023.1155666

**Published:** 2023-06-19

**Authors:** Lanxin Qiao, Yaxin Wang, Yi Liang, Tian Xia, Ling Li, Wei Xiong, Bin Liu, Yifan Feng, Yan Liu, Xu Jin, Jianmin Zhang

**Affiliations:** ^1^ Department of Anesthesiology, Beijing Children's Hospital, Capital Medical University, Beijing, China; ^2^ Department of Anesthesiology, Beijing Tiantan Hospital, Capital Medical University, Beijing, China

**Keywords:** inadvertent perioperative hypothermia, active warming strategy, children, temperature, randomized controlled trial

## Abstract

**Introduction:**

Inadvertent perioperative hypothermia (IPH) refers to a core body temperature lower than 36.0 °C, which can contribute to many adverse events. The special physiological characteristics in children further increase the incidence of IPH. Therefore, it is very important to perform effective perioperative warming measures for children. Traditional passive warming measures with additional layers have limited thermal insulation effects. Active warming measures might be the better choice, and most measures have achieved good effects in adults. This study combines a variety of active warming measures to propose perioperative active warming strategies and aims to verify the feasibility and thermal insulation effects of perioperative active warming strategies in children.

**Methods:**

This study is a multicentre, prospective, randomized controlled trial. From August 2022 to July 2024, 400 paediatric patients undergoing elective surgery will be recruited in four centres and randomly divided into the active warming strategies group and control group at a ratio of 1:1. The primary outcome is the perioperative cumulative hypothermia effect value (*Σ Δ*Ti × *Δ*ti, i = 1…, *n*). Multiple complications covering the anaesthesia recovery period and postoperative hospitalization will be considered as secondary outcomes to comprehensively analyse the prognosis.

**Trial registration:**

ClinicalTrials.gov identifier: ChiCTR2200062168. Registered on July 26th, 2022. Registered with the name of “Perioperative Active Warming Strategies in Children: A multicenter, prospective, randomized controlled trial”. URL:http://www.chictr.org.cn/showproj.aspx?proj=172778.

## 1. Introduction

Constant core body temperature is a necessary condition to ensure basic metabolism and vital activity, and normal core body temperature is generally between 36.5°C and 37.5°C (1, 2). A perioperative core body temperature lower than 36.0°C is defined as inadvertent perioperative hypothermia (IPH) (3). The incidence of IPH in adults is approximately 50% to 90% (4). IPH can contribute to a variety of perioperative complications (5), such as cardiovascular accidents (6, 7), increased bleeding (8, 9), surgical incision infection (10), delayed recovery (11), internal environment disturbance (12), and prolonged length of stay. Young age is an important risk factor for IPH (13, 14). The special physiological characteristics of children determine that their body temperature is more susceptible to environmental changes (15). Without proper warming measures, the incidence of IPH could be higher than that in adults. The thermoregulation system of children is not mature, including limited hair, less subcutaneous fat, and a greater overall surface area to body weight ratio, and the ATP-based independent thermoregulation mechanism has not been fully established, leading to insufficient heat production and excessive heat dissipation (5, 16). This relationship is especially true for infants, who generate heat through brown adipose tissue metabolism instead of shivering in low-temperature environments. This metabolism is innervated by sympathetic nerves and inhibited during general anaesthesia, further increasing the risk of IPH (17–19). Therefore, it is imperative to undertake appropriate warming measures for children during the perioperative period.

There have been growing concerns about perioperative complications caused by IPH, and passive warming measures have been widely used in perioperative anaesthesia management. However, a single layer of the passive warming measure can only reduce skin heat loss by approximately 30%, and adding additional layers can only barely increase benefits (20). In contrast, active warming strategies are more effective, and the effects have been verified in adult patients (21). Active warming strategies generally include forced-air warming blankets (19, 22), infusion heating apparatuses (23), forced air warmers (24), body cavity lavage liquid heating (25), increasing operating room temperature (26), etc. In addition, some studies have pointed out that prewarming 30–60 min before the operation can effectively decrease the incidence of IPH by reducing the redistribution of core body temperature (27, 28). The aforementioned active warming measures have been partially applied in children's perioperative management (29), but few trials have systematically studied the effects of the series of active warming strategies in children. This multicentre, prospective, randomized controlled trial aims to verify the effect of perioperative active warming strategies in children, further reducing the incidence of IPH and improving the prognosis in paediatric patients.

## 2. Materials and methods

### 2.1. Trial design

**Figure 1:**
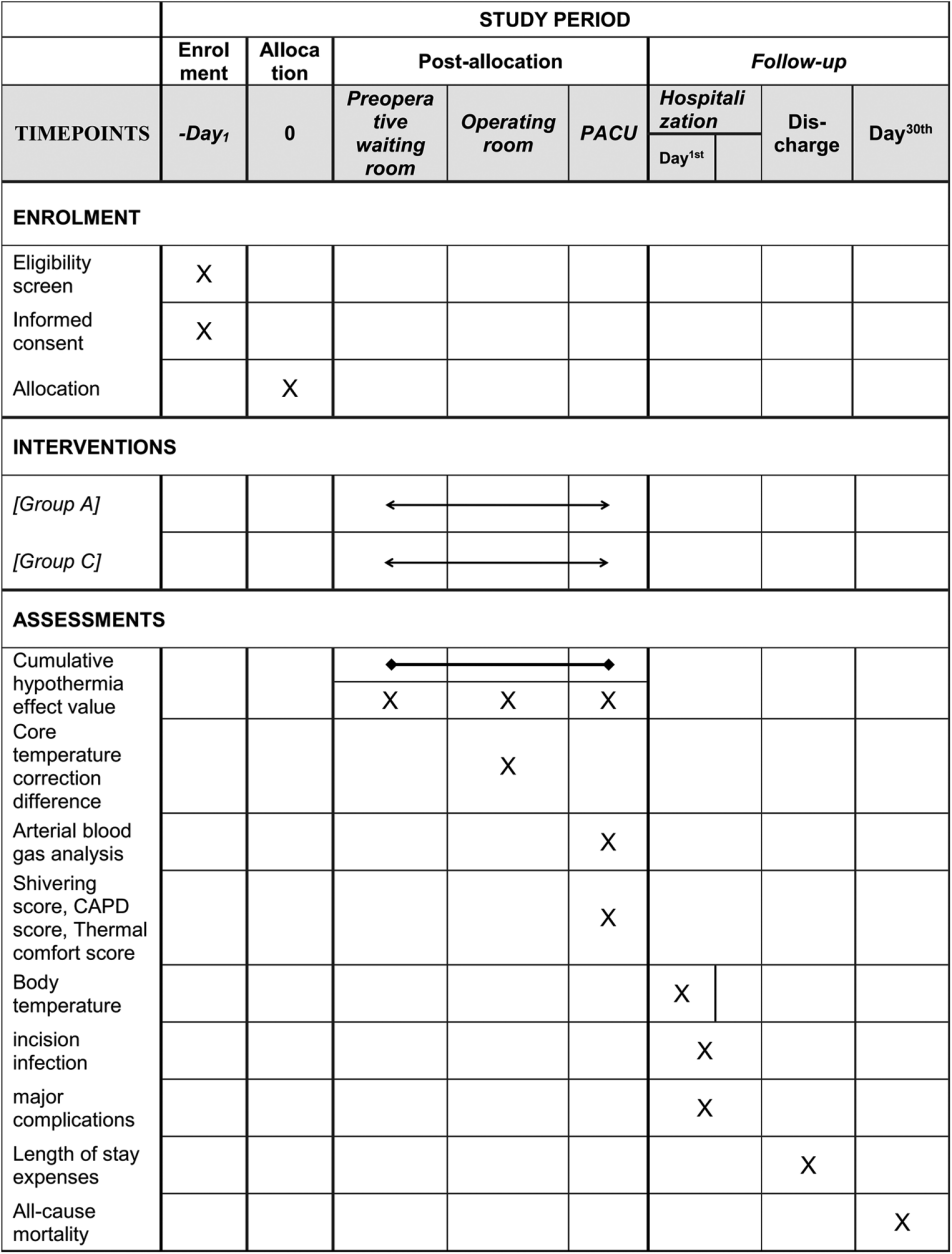
Study schedule. Group A, active warming strategies; Group C, control group; PACU, postanaesthesia care unit; CAPD, Cornell Assessment of Pediatric Delirium.

**Figure 2:**
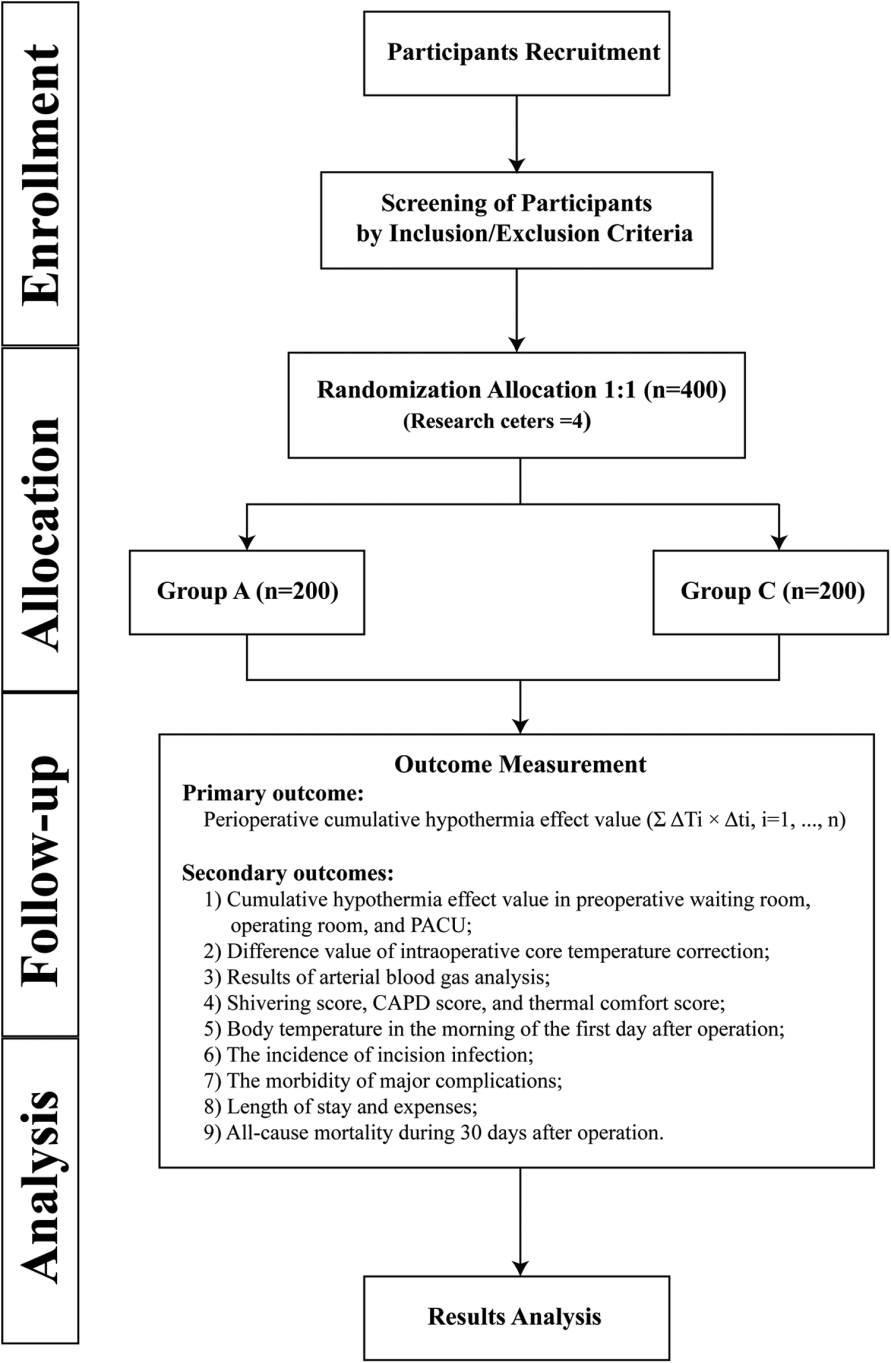
Patient flow diagram. Group A, active warming strategies; Group C, control group; PACU, postanaesthesia care unit; CAPD, Cornell Assessment of Pediatric Delirium.

This study is a multicentre, prospective, randomized controlled trial that will enrol 400 paediatric patients who meet the inclusion and exclusion criteria. The main research centre is Beijing Tiantan Hospital, Capital Medical University, and three other research centres will be recruited to assist in completing the study. The study schedule is shown in Figure 1, and the patient flow diagram is presented in Figure 2.

### 2.2. Ethics approval and consent to participate

This trial was approved by the Institutional Review Board (IRB) of Beijing Tiantan Hospital, Capital Medical University on July 3rd, 2022 (approval number: KY2022-101-02), and strictly adhered to the principles of the Declaration of Helsinki. All eligible paediatric patients and their legal guardians will be informed of the trial protocol within the period from the first day of hospitalization to the day before the operation and will be given sufficient time to consider whether to participate. The paediatric patients will be formally included in this trial only if their legal guardian (and school-age children) sign the informed consent form, and they will be able to withdraw at any time during the study period.

We also attach great importance to the information security of participants. Each child involved in this trial will be assigned a unique identification code to hide his or her private information after signing the informed consent form, and the code will not be shared outside this study. All paper materials, such as case report forms (CRFs), will be locked in a cabinet of the anaesthesiology department of Beijing Tiantan Hospital, Capital Medical University, and the electronic information will be stored in EpiData software (version 3.1 data entry). Only the principal investigator (PI) and IRB have access to all of the information. The researchers in charge of the results analysis will need to apply for access permission from the PI, and the access time will be recorded in detail.

### 2.3. Eligibility

Children aged 1–12 years old scheduled for elective surgery will be screened for eligibility. The inclusion and exclusion criteria are as follows.

#### 2.3.1. Inclusion criteria

(1) Paediatric patients ≥1 year old and ≤12 years old, no gender limitations;
(2) Scheduled for elective non-cardiac and non-transplant surgery under general anaesthesia or general anaesthesia combined with other anaesthesia methods (The specific types of surgery include pediatric general surgery, pediatric neurosurgery, pediatric urological surgery, pediatric otorhinolaryngologic surgery surgery);
(3) American Society of Anesthesiologists physical status of I–II;
(4) Estimated operation time of >40 min; and
(5) Growth according to the normal range of WHO Child Growth Standards.

#### 2.3.2. Exclusion criteria

(1) Abnormal body temperature caused by diseases or medicines before surgery;
(2) Elective surgery requiring controlled hypothermia;
(3) Children with a definite history of cardiac insufficiency or congenital heart disease;
(4) Children with a definite history of metabolic diseases, such as thyroid dysfunction and diabetes;
(5) Changes in laboratory examination of routine blood, blood biochemistry, and blood clotting exceeding 1.5 times the normal value;
(6) The tracheal tube expected to be retained after the operation;
(7) Postoperative thermoregulation expected to be affected, such as the neurosurgery in the vicinity of the hypothalamic thermoregulatory centre; and
(8) Unwillingness to participate in this trial or participate in other clinical trials.

#### 2.3.3. Dropout criteria

(1) Necessary postoperative sedation therapy;
(2) Retaining of tracheal tube accidentally after operation;
(3) Receiving a second operation during hospitalization; and
(4) Refusal to continue to participate in the experiment.

### 2.4. Intervention

A total of 400 children will be randomly assigned to the active warming strategies group and the control group. The specific warming measures are as follows.

**Figure 3:**
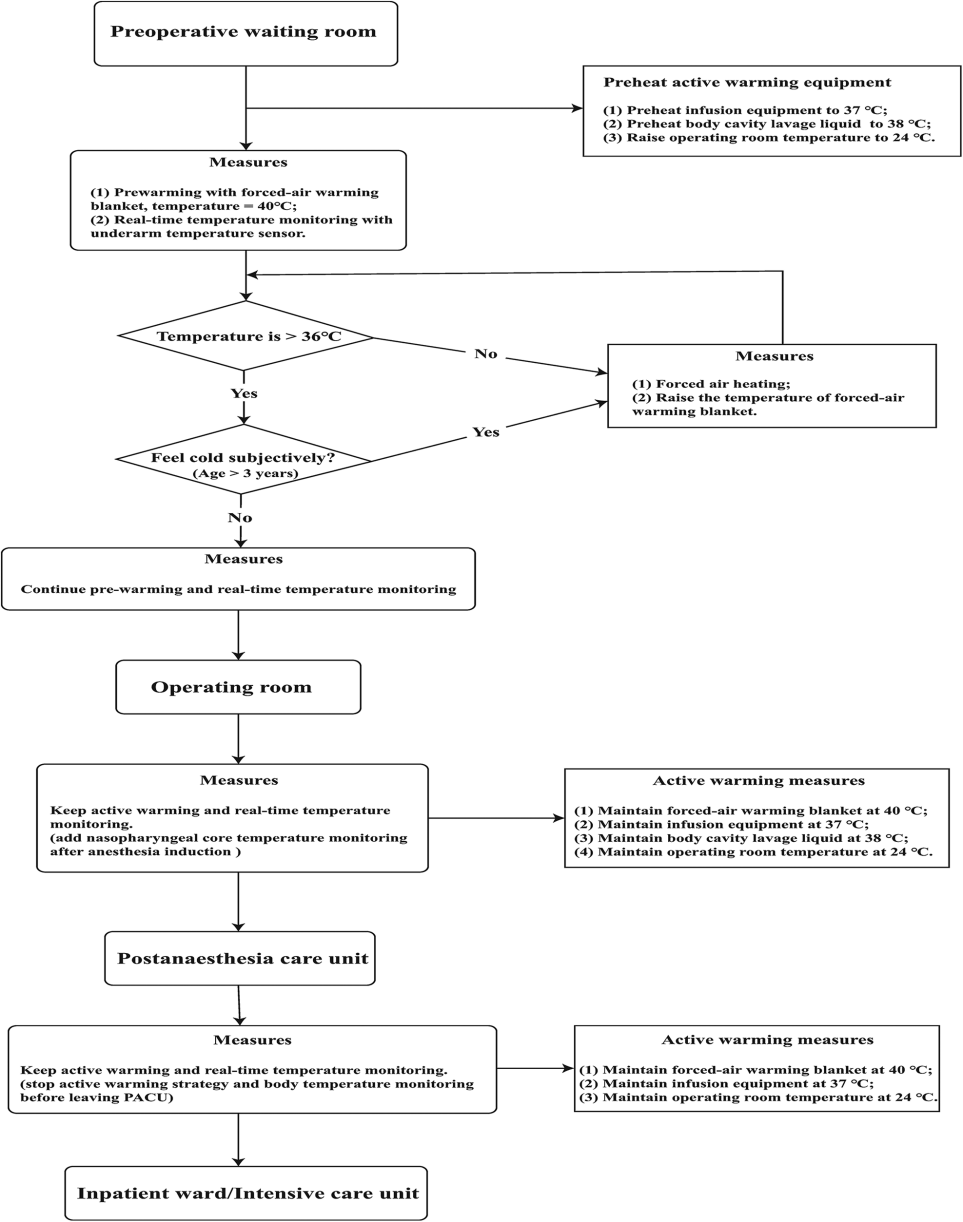
Active warming strategies process.

**Active warming strategies group (Group A):** Details about the active warming strategy are mainly described in Figure 3, which was carried out in a total of three locations, including the preoperative waiting room, the operating room and the postanaesthesia care unit (PACU). In the preoperative waiting room, pre-warming is mainly carried out using a forced-air warming blanket (40°C). If the child's body temperature is <36°C or subjectively feels cold, we further raise the temperature of the forced-air warming blanket and the temperature of the waiting room. Active warming measures in the operating room are to maintain the forced-air warming blanket at 40°C, the infusion equipment at 37°C, the body cavity lavage liquid at 38°C and the operating room temperature at 24°C. Active warming measures in the PACU are to maintain the forced-air warming blanket at 40°C, the infusion equipment at 37°C and the room temperature at 24°C. Real-time temperature monitoring will be maintained throughout the whole process. The nasopharyngeal core temperature will be monitored additionally for correction after anaesthesia induction and before awakening. All active warming measures will be discontinued on exit from the PACU.

**Control group (Group C):** Routine passive warming measures will be used in the perioperative period. Children will be covered in insulation layers from entering the preoperative waiting room to leaving the PACU. In the preoperative waiting room, the children will still use a warming blanket but not heated forced air and not raise the room temperature. In the operating room, the warming blanket is not heated and the rest of the warming measures, including the infusion equipment, the body cavity lavage liquid, and the temperature of the operating room, is at the discretion of the anaesthesiologist in charge. In the PACU, the warming blanket is also not heated and the rest of the warming measures, including the infusion equipment, and the temperature of the operating room, is at the discretion of the anaesthesiologist in charge. Similarly, real-time temperature monitoring will be maintained throughout the whole process. The nasopharyngeal core temperature will be monitored additionally for correction after anaesthesia induction and before awakening. If the children's temperature is <35°C throughout the perioperative period, regulations for body temperature protection will be enforced as described below.

**Regulations for body temperature protection:**

(1) If the core temperature of children in the two groups is <35°C, the active warming measures will be forced to start, or the warming level will be improved to avoid hypothermia.
(2) If the children in Group A complain that they are hot, or the body temperature >37.5°C, the temperature of the forced-air warming blanket will be adjusted down until the children feel comfortable, or the body temperature is ≤37.5°C.

In addition, the intervention measures involved in this study are routine clinical treatments, with little risk. It is expected that no damage will be caused to the participants, and there is no relevant compensation agreement. Children will be monitored routinely throughout the perioperative period and if there are serious adverse events related to this study during the study, such as cardiac arrest caused by severe abnormal body temperatures, they will be reported immediately to the PI and IRB by the anaesthesiologist in charge to break the blinding or terminate the study as appropriate.

### 2.5. Randomization and blinding

This study applies a randomized stratified block design. According to the number of research centres, it is divided into four strata. In each stratum, the number of blocks is 2, and the length of the blocks is 4. The eligible children will be randomly assigned to Group A and Group C at a ratio of 1:1. The randomization will be completed by SAS software (SAS Institute Inc., USA). The allocation information will be concealed in sealed, opaque envelopes and opened by the anaesthesiologists in charge before the children enter the preoperative waiting room. To ensure the participants' safety and to complete warming measures, the anaesthesiologists in charge will not be blinded, and they will not participate in follow-up or results analysis. In addition, the allocation information will be blinded for patients and the researchers in charge of follow-up. The interim analysis will be completed by researchers independent of the overall trial process.

### 2.6. Standardized anaesthesia management

All eligible children will receive standardized anaesthesia management procedures. Except for real-time axillary temperature monitoring, when the children enter the operating room, standard monitoring will be applied, including noninvasive blood pressure, heart rate, and pulse oximetry saturation. Invasive arterial pressure, end-tidal carbon dioxide partial pressure, anaesthesia gas monitoring, and the nasopharyngeal core temperature will be monitored after anaesthesia induction. Before anaesthesia induction, the anaesthesiologist in charge intravenously will administer midazolam (0.025–0.075 mg·kg^−1^). General anaesthesia will be induced with sufentanil 0.5 μg·kg^−1^, propofol 2–3 mg·kg^−1^, and cis-atracurium 0.1–0.2 mg·kg^−1^ or rocuronium 0.4–0.6 mg·kg^−1^. After intubation, mechanical ventilation will be set in the volume-controlled mode. The tidal volume will be 8–10 ml·kg^−1^ and the respiratory rate will be 14–20 breaths per minute. Remifentanil (0.1–0.2 μg·kg^−1^·min^−1^) and propofol (8–10 mg·kg^−1^·h^−1^) will be used to maintain the total intravenous infusion anaesthesia and adjusted for sedative and analgesic needs during the operation. Corresponding treatment will be administered according to the children's status to maintain the mean arterial pressure and heart rate within 30% of the baseline values intraoperatively. The infusion of propofol and remifentanil will be stopped at the end of surgery. After extubation, children will be transferred to the PACU and further transferred to the inpatient ward or intensive care unit based on their condition.

### 2.7. Outcome measurement

#### 2.7.1. Primary outcome

Perioperative cumulative hypothermia effect value (*Σ Δ*T_i_ × *Δ*t_i_, i = 1…, *n*).

Note: When the body temperature at a certain time (i) is <36°C, hypothermia variation will be recorded as *Δ*T_i,_ (*Δ* T_i _= 36°C-T_i_), and the duration of this hypothermia variation will be recorded as *Δ*t_i_.

#### 2.7.2. Secondary outcomes

(1) Cumulative hypothermia effect value (*Σ Δ*T_i_ × *Δ*t_i_, i = 1…, *n*) in the preoperative waiting room, operating room, and PACU;
(2) Difference value between intraoperative core temperature and axillary temperature;
(3) Results of arterial blood gas analysis in the anaesthesia recovery period, including pH, lactic acid, and haemoglobin values;
(4) Shivering score, Cornell Assessment of Pediatric Delirium score, and thermal comfort score in the anaesthesia recovery period;
(5) Body temperature in the morning of the first day after surgery;
(6) The incidence of incision infection during hospitalization;
(7) The morbidity of major complications during postoperative hospitalization;
(8) Length of stay and expenses;
(9) All-cause mortality 30 days after the operation.

Note: a. Anaesthesia recovery period refers to the time from stopping anaesthetic infusion to the time when the children are completely conscious and fully awake. b. Major complications during postoperative hospitalization include postoperative pulmonary complications, arrhythmias requiring treatment, myocardial infarction, sepsis, renal failure, central nervous system events, anastomotic leakage, and accidental transfer to the intensive care unit.

#### 2.7.3. Other data to be recorded

**Preoperative:** Baseline data, including demographic characteristics, basic vital signs, complications, and information related to anaesthesia and operation.

**Intraoperative:** Record vital signs and respiratory parameters every 10 min during anaesthesia, the type and dose of intraoperative medicines, intraoperative adverse events, total fluid volumes, and the durations of anaesthesia, surgery, and the recovery period.

**Postoperative:** The PACU stay period, medicine administration, Ramsay Sedation score and Wong-Baker Faces Scale scores recorded when the patient leaves the PACU.

**Follow-up:** Will be conducted during postoperative hospitalization and on the 30th day after the operation.

### 2.8. Sample size calculation

This study is a multicentre, prospective, randomized controlled trial. According to the pretest, the mean ± standard deviation of the perioperative cumulative hypothermia effect value (*Σ Δ*T_i_ × *Δ*t_i_, i = 1…, *n*) under passive warming measures is 12.2 ± 4.31, and using perioperative active warming strategies, it can be decreased by 15%. Considering a dropout rate of 20%, 400 subjects will be required with 90% power and an *α* level of 0.05. The sample ratios of the four centres are *Q*_1 _= *Q*_2 _= *Q*_3_= *Q*_4 _= 1/4N = 100.

### 2.9. Interim analysis

This trial will conduct interim analysis when the sample size reaches 50% to compare the effect and safety between the two groups by preserving the sample size as much as possible. The interim analysis will be supervised by the independent data management committee (DMC). Except for the researchers directly involved in the interim analysis, other researchers in this trial will still be blinded to the allocation information. The trial will use the Lan-DeMets alpha spending function with an O'Brien-Fleming boundary to correct the test level of interim analysis, and the effective test level to terminate the trial is *p* < 0.003.

### 2.10. Stratified analysis

Owing to the significant differences in age and growth among the included children, we will conduct stratified analysis on toddlers aged 1–3, preschool children aged 3–6, and school-age children aged 7–12 to obtain the response of each age stratum to active warming strategies as much as possible.

### 2.11. Statistical analysis

Statistical analysis will be performed by SPSS software, version 24. (International Business Machines Inc., USA). Continuous variables will be reported as the mean ± standard deviation ($\bar{x}\pm s$) or medians and interquartile ranges. Categorical variables will be presented as numbers (proportion, %). The Kolmogorov-Smirnov test will be performed to detect the normal distribution of continuous variables. Student's t test will be used for normally distributed continuous variables, and the Kruskal-Wallis H test will be used for abnormally distributed data. The grade variables will be analysed using the Mann-Whitney U test. The chi-square test and Fisher's exact test will be used to analyse categorical variables. Repeated measures analysis of variance will be applied for the analysis of repeated measures data. *p* < 0.003 is defined as statistically significant in interim analysis, and the statistical significance of the final analysis is set at *p* < 0.05.

## 3. Discussion

This trial aims to verify the feasibility and thermal insulation effect of perioperative active warming strategies by comparing them with traditional passive warming measures to further improve the prognosis of paediatric patients. The primary outcome is the perioperative cumulative hypothermia effect value (*Σ Δ*T_i_ × *Δ*t_i_, i = 1…, *n*), which is chosen because it can better quantify the level of IPH and can more effectively reflect the differences between the two groups. In addition, we combine multiple secondary outcomes to comprehensively analyse the prognosis, which will provide more clinical evidence about active warming strategies.

This study also has some limitations. First, we do not limit the surgery to a single category, which could be a confounding factor. However, considering the desire to validate the generalisability of the active warming strategy in this study, we wanted to cover a relatively wide range of types of paediatric procedures. Second, the population included in this study is children aged 1–12 years, who have a wide variation in age and growth. A stratified analysis will be conducted on children in different age levels. Third, this trial will perform perioperative real-time temperature monitoring with an axillary temperature sensor. Although the core body temperature is closer to the real temperature, awake children might not tolerate this method of temperature monitoring. Axillary temperature monitoring is relatively accurate and easy to tolerate, and we can additionally measure the nasopharyngeal core body temperature after anaesthesia induction for correction.
